# Assessment of Systemic and Cerebral Oxygen Saturation during Diagnostic Bronchoscopy: A Prospective, Randomized Study

**DOI:** 10.1155/2020/8540350

**Published:** 2020-12-09

**Authors:** Attila Vaskó, Sándor Kovács, Béla Fülesdi, Csilla Molnár

**Affiliations:** ^1^Department of Pulmonology, University of Debrecen, Faculty of Medicine, Health and Medical Science Centre, Debrecen, Hungary; ^2^Institute of Sectoral Economics and Methodology, Faculty of Economics and Business, Department of Research Methodology and Statistics, University of Debrecen, Debrecen, Hungary; ^3^Department of Anesthesiology and Intensive Care, University of Debrecen, Faculty of Medicine, Health and Medical Science Centre, Debrecen, Hungary

## Abstract

**Background:**

Arterial hypoxemia occurs in about 2.5–69% of cases during fiberoptic bronchoscopy and may necessitate administration of supplemental oxygen. Whether routine supplementary administration is indicated for all patients is a debated issue. In this prospective randomized study, we assessed the incidence of systemic desaturation (SpO_2_ <90% or *a* >4% decrease lasting for more than 60 s) and wanted to find out whether cerebral desaturation occurs in parallel with systemic changes.

**Patients and Methods:**

92 consecutive patients scheduled for diagnostic bronchoscopy were randomly assigned to the no oxygen (O_2_- group), 2 l/min supplemental O_2_, or 4 l/min supplemental O_2_ groups. Primary end points were systemic and cerebral desaturation rate during the procedure. Secondary end points were to delineate the main risk factors of systemic and cerebral desaturation.

**Results:**

In the entire cohort, systemic desaturation occurred in 18.5% of patients (*n* = 17), corresponding to 5 patients (16%) in the O_2_ (−)group, 6 patients (19%) in the 2 l/min group, and 6 patients (20%) in 4 l/min group, respectively. In the O_2_ (−) group, the probability of desaturation was 41.7 times higher than that in the 2 l/min group (*p*=0.014 s), while there was no difference in the probabilities of desaturation between the 2 l/min and 4 l/min groups (*p*=0.22). Cerebral desaturation (more than 20% rSO_2_ decrease compared to baseline) did not occur in any patients in the three groups. Systemic desaturation developed earlier, and recovery after desaturation was longer in the O_2_ (−) group. Male gender, smoking, and systemic oxygen saturation at baseline and FEV1% were the most significant factors contributing to systemic desaturation during bronchoscopy.

**Conclusions:**

Administration of supplemental oxygen does not prevent systemic desaturation during flexible bronchoscopy, but may contribute to the shortening of desaturation episodes and faster normalization of oxygen saturation. According to our results, 2 l/min supplemental oxygen should routinely be administered to patients throughout the procedure. This trial is registered with NCT04002609

## 1. Background

Fiberoptic bronchoscopy remains an essential tool in the evaluation of pulmonary diseases. Arterial hypoxemia might be one of the most important side effects of the intervention occurring in 2.5–69% of cases and necessitating administration of supplemental oxygen [1, 2]. Accordingly, the guideline of the British Thoracic Society recommends routine monitoring of oxygen saturation during bronchoscopy. Routine administration of supplemental oxygen for all patients undergoing fiberoptic bronchoscopy is still a debated issue [1]; therefore, the guideline recommends the use of oxygen supplementation only to achieve an oxygen saturation of at least 90% [3].

Despite previous studies, not all details of the predisposing factors of desaturation during the procedure are known. Some investigators found that FEV1% and an obstructive pattern of pulmonary function tests are the most important clinical factors for the prediction of hypoxemia, while others could not prove this relationship [4]. In addition, the duration of desaturation episodes may vary widely, despite supplemental oxygen administration [4]. According to a retrospective analysis of Sinha et al., severe desaturation necessitating early termination of bronchoscopy occurs in 2.4% of cases [5]. At present, it is not known whether these systemic desaturation episodes might influence the oxygen saturation of the most sensitive organ, the brain tissue.

In line with the above, we conducted a prospective, randomized study that aimed to answer the following questions:

- What is the incidence of systemic and cerebral desaturation in patients undergoing fiberoptic bronchoscopy and receiving no supplemental oxygen and two different oxygen supplementations (2 l/min and 4 l/min)?
- Is there a relationship between systemic desaturation episodes and cerebral desaturation?
- What are the main risk factors of systemic and cerebral desaturation in patients undergoing fiberoptic bronchoscopy?

## 2. Patients and Methods

Consecutive patients between the time frame of January 2018 to June 30 admitted to the Bronchology Laboratory of the Department of Pulmonology, University of Debrecen, for flexible bronchoscopy were asked to participate in the study. The study was approved by the Medical Ethics Committee of the University of Debrecen (registration number: 4989-2018) and was registered on ClinicalTrials under the number of NCT04002609. After being given a detailed explanation of the procedure, all patients gave written informed consent.

The indication of flexible bronchoscopy was based in all cases on the results of medical history, physical examination, chest X-ray and/or chest CT scan, lung function tests, and laboratory parameters including hemoglobin concentration and hemostatic variables as well as blood gas analysis when necessary. Bronchoscopy was performed in all cases with suspected lung cancer for the purpose of cytological or histological sampling.

Bronchoscopy was performed using the PENTAX EB-1975K (Pentax Medical, Hamburg, Germany) device after a fasting period of at least 4 hours. The procedure was performed in the supine position after topical administration of lidocaine 2% solution. Routine monitoring consisted of ECG, noninvasive blood pressure measurement, and pulse oximetry (finger probe). As an additional monitoring tool, a near-infrared monitoring sensor was placed on the forehead of patients' dominant hemisphere for monitoring cerebral oxygen saturation. An INVOS 5100C cerebral oximeter (Covidien LLC, 15 Hampshire Street, Mansfield, MA 02048, USA) was used for cerebral near-infrared spectroscopy measurements.

### 2.1. Patient Grouping

Patients undergoing bronchoscopy were randomly assigned (presealed envelope randomization) to three different groups as follows:

  Group A (*N* = 31 patients): patients in this group did not receive any oxygen supplementation during the procedure. Rescue supplemental oxygen through nasal cannula was provided if clinically significant desaturation could be observed during bronchoscopy. Significant desaturation was defined as systemic oxygen saturation ≤90% on pulsoxymetry or a relative change of ≥4% lasting for ≥1 minute. Cerebral desaturation was defined as a more than 20% decrease in rSO_2_ compared to baseline measured using near-infrared spectroscopy.
  Group B (*N* = 31 patients): supplemental oxygen was provided for the patients through a nasal cannula by a flow rate of 2 l/min throughout the procedure.
  Group C (*N* = 30 patients): supplemental oxygen was administered through a nasal cannula by a flow rate of 4 l/min throughout the procedure.

The following data were collected or calculated in all patients prior to bronchoscopy for the sake of later analysis:

- Hemoglobin concentrations (g/L)
- FVC % = forced vital capacity (%)
- FEV1% = forced expiratory volume for 1 second expressed as a percentage
- Tiffeneau index = FEV1/FVC
- Parameters registered or calculated during the bronchoscopy procedure are as follows:
- Pulse rate
- Systemic oxygen saturation using finger probe pulsoxymetry
- Cerebral tissue oxygen saturation

### 2.2. Study End Points

1. Primary end point was defined as the incidence of systemic and cerebral desaturation in the three groups
2. Secondary end points were factors influencing systemic and cerebral desaturation

### 2.3. Statistical Analysis

#### 2.3.1. Power Analysis

As a first step, we performed a power analysis for determining the sample size. Based on our pilot study performed among ten patients, we observed a 3.1 ± 1.2 decrease in systemic oxygen saturation during bronchoscopy without oxygen administration. Based on this, we hypothesized that administration of 4 l/min oxygen through a nasal cannula results in a less than 1% decrease in systemic oxygen saturation. Using an alpha of 0.05 and a power of 90%, the necessary number of patients to be included was calculated as 30 per group. With a further “Apriori” power analysis, the required sample size for a one-way independent ANOVA analysis of “systemic O2” within the 3 study groups was calculated. The effect size (ES) in this study was considered large using Cohen's criteria [6]. With alpha = 0.05, power  = 0.9, and ES = 0.4, the projected sample size per group was approximately *N* = 27.39 using the power calculator of Australia and New Zealand Melanoma Trial Group [7]. Thus, our proposed total sample size of 92 for the 3 groups (30+ samples per group) can be considered adequate for the major objectives of our study. Furthermore, a “sensitivity” power analysis was also performed with a total sample size of 92, an average “systemic O2” of 97.23, and a relative variance of 2.6%. We obtained a very large actual effect size ES = 0.506 in our analysis.

Before starting statistical analysis, parameters in all groups were checked for normality by the Kolmogorov–Smirnov test. For normally distributed data, the *t*-test was used, whereas in the case of nonnormal distribution, the ANOVA test was used. Pearson correlation was applied for testing the relationship between systemic and cerebral oxygen saturations.

#### 2.3.2. Hurst Exponent Calculations for Checking the Stability of O_2_ Saturation

In order to check the changes in systemic and cerebral oxygen saturation during bronchoscopy, we applied the Hurst exponent calculations that indicate the probability of desaturation during the entire procedure. The more the Hurst exponent exceeds 0.5, the lower is the probability of desaturation throughout the procedure. In addition, higher values of the Hurst exponent refer to a stable trend of oxygen saturation [8].

#### 2.3.3. Assessment of Factors of Desaturation

The Cox multiparametric proportional hazard model was used for assessing the underlying factors of systemic and cerebral desaturation. The following parameters were considered as continuous parameters: gender, smoking, hemoglobin concentration, FVC%, FEV1%, and mean systemic O_2_ saturation. A-C groups were considered as categorical variables in the multiparametric model.

## 3. Results

Demographic parameters are summarized in Table 1. In the total cohort, the mean age was 61.9 ± 12.7 years, with a female-male ratio of 40 : 60%. There were no differences between smokers and nonsmokers between females and males. However, females showed significantly higher preprocedural FVC% and FEV1% values.

### 3.1. Primary End Points

**Figure 1:**
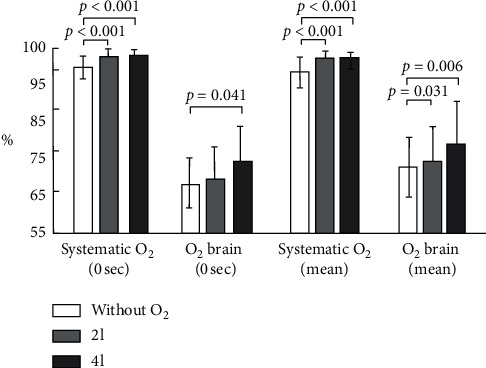
Absolute values of systemic and cerebral oxygen saturation at baseline (0 sec) and averaged values during bronchoscopy (mean). Means and standard deviations are shown. 2 l and 4 l indicate 2 l/min and 4 l/min supplemental oxygen administration.

Average systemic and cerebral oxygen saturations before and during bronchoscopy are shown in Figure 1. It is obvious from the figure that administration of any supplemental oxygen (2 l/min or 4 l/min) results in a significant improvement of both systemic and cerebral oxygen saturation. However, there were no differences in oxygen saturation values between the 2 l/min and the 4 l/min supplemental oxygen group.

In the entire cohort, systemic desaturation occurred in 18.5% of the patients (*n* = 17). This corresponds to 5 patients (16%) in the O_2_ (−)group, 6 patients (19%) in the 2 l/min supplemental O_2_ group, and 6 patients (20%) in the 4 l/min supplemental oxygen group, respectively. The number of desaturations in the different groups did not reach the level of statistical significance (chi-square: 0.17; *p*=0.91). It has to be mentioned that detailed statistical analysis indicated that, in the O_2_-negative group, the probability of desaturation is 41.7 times higher than that in the 2 l/min supplemental oxygen group (*p*=0.014), while there was no difference in the probabilities of desaturation between the 2 l/min and 4 l/min supplemental oxygen groups (*p*=0.22).

Cerebral desaturation (more than 20% decrease in rSO2 compared to the baseline) did not occur in any patient in the three groups during the bronchoscopy procedure. There was no significant relationship between systemic desaturation and cerebral oxygen saturation as measured by near-infrared spectroscopy (Pearson correlation coefficient: −0.07).

### 3.2. Secondary End Points

#### 3.2.1. The Effect of Gender

**Figure 2:**
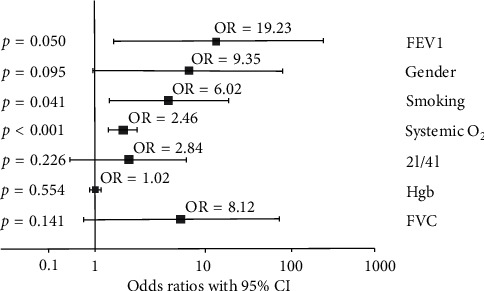
The effect of confounding factors on systemic desaturation.

Desaturation occurred in 22.2% of males, while it was observed in 13.2% of females. Men had a 9.3 times greater chance to develop systemic desaturation during the procedure than women, irrespective of supplemental oxygen administration (Figure 2).

**Figure 3:**
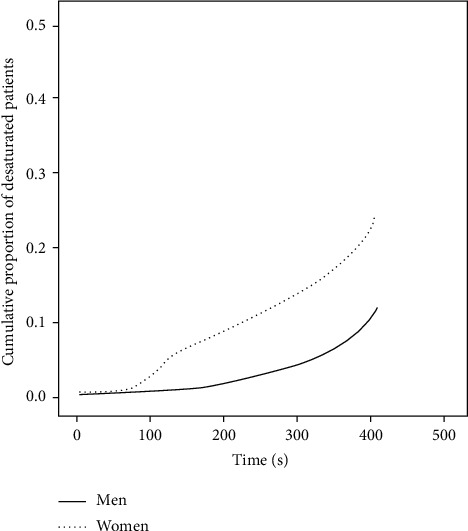
Cumulative proportion of systemic desaturations in female and male patients.

The time that elapsed between starting bronchoscopy and desaturation was 207 ± 111 sec for males and 226 ± 138.3 sec for females (*p* < 0.01).  Figure 3 demonstrates the cumulative proportion of systemic desaturations in female and male patients.

Based on a more detailed statistical analysis, in females, systemic oxygen saturation was stable throughout the procedure only if 4 l/min supplemental oxygen was administered (Hurst exponent below 0.5), whereas in males, 2 l/min supplemental oxygen resulted in stable systemic oxygen saturation during the entire course of the bronchoscopy procedure (Table 2.)

#### 3.2.2. Smoking

**Figure 4:**
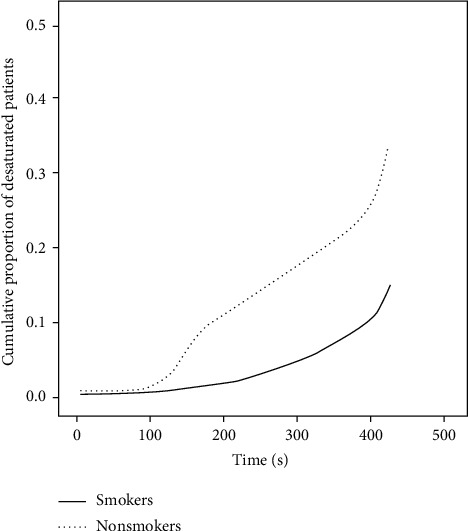
Cumulative proportion of systemic desaturations in smokers and nonsmokers.

Desaturation occurred in roughly 20% of smokers, while it was observed in only 14% of nonsmokers. The risk of desaturation at any time point of the bronchoscopy procedure was 6-fold higher in smokers than in nonsmokers (Figure 2). In general, desaturation occurred in smokers after 194 ± 114.6 seconds and in nonsmokers after 240 ± 120.8 seconds.  Figure 4 depicts the cumulative proportion of systemic desaturations in smokers and nonsmokers.

#### 3.2.3. Other Factors

Systemic desaturation was not influenced by hemoglobin concentrations. Based on the analysis, FEV1% was a significant determining factor in the development of desaturation (Figure 2). We found that every 100 ml change in FEV1 results in 50% improvement in the risk of systemic desaturation during bronchoscopy. In contrast to this, FVC % did not have a significant impact on systemic desaturation. Systemic desaturation was also independent of age: the age of patients showing desaturation was 60.4 ± 15.5 years vs. nondesaturation patients, 62.2 ± 12.1 years; *p*=0.591.

## 4. Discussion

In this prospective, randomized study, we found that systemic desaturation occurs in 18.5% of patients, despite supplemental oxygen therapy. It should be noted that supplemental oxygen improved both systemic and cerebral oxygen saturation during the procedure and patients who did not receive oxygen supplementation had a 41.7-fold higher risk for systemic desaturation. Another main finding of the present study is that systemic desaturation did not become manifested in the cerebral tissue.

Systemic desaturation during bronchoscopy was described as far back as 40 years ago by Albertini et al. [9]. Its incidence varies between 2.5 and 69%, depending on the definition (threshold SpO2 and duration of desaturation), even if no sedation is used [1, 10]. According to the observations of Golpe and Mateos, desaturation (defined as SpO2 < 90%) occurred in 69% during and in 72% after bronchoscopy [1]. In the study of Jones et al., desaturation (SpO2 < 90%) was observed in 24% of cases, but lasted for 20–30 s only in 14.4% [4]. Alijanpour et al. administered supplemental oxygen only for patients who experienced an SpO_2_ value of <90% and found that it was necessary in 5.5% of cases [11]. In a recent study of Pertzov et al., it was shown that, during bronchoscopies performed under light midazolam-fentanyl sedation, desaturation rate may be close to 90% [12]. In our study, desaturation (SpO_2_ < 90% and/or a >4% decrease, lasting for more than 60 seconds) occurred in 16%, 19%, and 20% of patients in the O_2_ (−), 2 l/min, and 4 l/min groups, respectively. Thus, despite supplemental oxygen administration, desaturation did occur in all groups. It should be noted, however, that systemic desaturation developed earlier and recovery after desaturation was longer in patients who did not receive O_2_ supplementation.

We found that male gender, smoking, systemic oxygen saturation at baseline, and FEV1% were the most significant factors that contributed to systemic desaturation during bronchoscopy. In contrast to our results, Fang et al. were unable to prove the determining role of male gender in desaturation [13]. In our cohort, it could be unequivocally demonstrated that desaturation is not only more frequent in males than females but occurs earlier, despite supplemental oxygen administration. Similarly, the risk of desaturation episodes was 6 times higher in smoking patients and desaturation developed earlier. Although we could not find previous studies proving these observations, an obstructive pattern on pulmonary function tests has been shown in previous studies as a predisposing factor of desaturation [1, 4, 11, 14] and the relation between smoking and obstructive disease is widely known [15–17]. The determining role of preprocedural FEV1 in the development of systemic desaturation has been documented in several studies [1, 11, 13, 14].

To the best of our knowledge, this was the first study to assess systemic and cerebral oxygen saturation in parallel during flexible bronchoscopy. Our main goal was to assess oxygen saturation in the organ that is most sensitive to hypoxemia, especially during desaturation episodes. In previous studies, near-infrared spectroscopy was effectively used for assessing cerebral tissue oxygen saturation during thoracic surgeries [18, 19]. In the present study, no significant desaturation occurred in the cerebral tissue during bronchoscopy, despite systemic desaturations. It is conceivable that short-term systemic desaturations are counteracted by the flow-metabolism coupling regulation of the brain tissue, preserving the brain tissue during short-term decreases of systemic oxygen saturation [20].

In conclusion, administration of supplemental oxygen does not prevent systemic desaturation during flexible bronchoscopy, but may contribute to a shortening of desaturation episodes and faster normalization of oxygen saturation. According to our results, 2 l/min supplemental oxygen should routinely be administered to patients throughout the procedure.

## Figures and Tables

**Table 1:** Demographic parameters and confounding factors.

	Total cohort	Males	Females	*p* value
Age (years)	61.9 (12.7)	62.8 (11.3)	60.5 (14.5)	NS
Smokers/non‐smokers (%)	48.9/51.1	48.1/51.9	50/50	NS
FVC%	85.52 (23.13)	79.70 (21.26)	93.79 (23.44)	0.003
FEV1%	76.34 (25.21)	71.00 (23.97)	83.92 (25.29)	0.015

Means and standard deviations are shown. NS indicates nonsignificant differences.

**Table 2:** Absolute values of systemic O_2_ saturation at baseline (0 sec syst. O_2_%) and during bronchoscopy (syst. O_2_mean %) and Hurst exponent values in males and females.

	Study group			Pairwise comparisons			ANOVA F statistic
	O_2_ (−)	2 l	4 l	O_2_ (−) vs. 2 l/min	O_2_ (−) vs. 4 l/min	2 l/min vs. 4 l/min	
	Males						
0 sec syst. O_2_ %	95.20	97.79	98.25	−2.59^*∗*^	−3.05^*∗∗*^	−0.46	9.52^*∗∗∗*^
Syst. O_2_mean %	93.93	96.92	97.05	−2.99^*∗∗*^	−3.12^*∗∗*^	−0.13	5.22^*∗∗∗*^
Hurst exp. syst. O_2_	0.48	0.59	0.75	−0.11	−0.27^*∗∗*^	−0.16^*∗*^	9.01^*∗∗∗*^

Females							
0 sec syst. O_2_ %	95.69	98.33	98.30	−2.65^*∗∗*^	−2.61^*∗∗*^	0.03	6.09^*∗∗*^
Syst. O_2_mean %	94.63	98.36	97.24	−3.73^*∗∗*^	−2.61^*∗*^	1.12	5.68^*∗*^
Hurst exp. syst. O_2_	0.42	0.49	0.70	−0.06	−0.27^*∗∗*^	−0.21^*∗∗*^	7.10^*∗∗*^

O_2_ (−) indicates no supplemental oxygen; 2 l and 4 l indicate 2 and 4 l/min supplemental oxygen. ^*∗∗∗*^ *p* < 0.001,^*∗∗*^ *p* < 0.01, and ^*∗*^ *p* < 0.05.

## Data Availability

Data are available from the corresponding author upon request.

## Abbreviations

FEV1: Forced expiratory volume in the first second
FVC: Forced vital capacity.
